# Character as Demanded in Professional Life

**Published:** 1893-07

**Authors:** Forrest G. Eddy

**Affiliations:** Providence, R. I.


### CHARACTER AS DEMANDED IN PROFESSIONAL LIFE.[1]

BY FORREST G. EDDY, D.M.D., PROVIDENCE, R. I.

It used to be the practice, and perhaps continues to be, in New England churches, for ministers, when the occasion required, or when matters had for some time been running perhaps too smoothly

---

[1] Read at the annual meeting of the Harvard Odontological Society, Boston, February 25, 1893.

in the ecclesiastical grooves, to deliver what was known as a "doctrinal sermon,"—one of those terrible appeals which were calculated to arouse the imaginations and the consciences of the most hardened listeners. It was a sort of old-fashioned way of "rounding up" the flock, and of branding anew the straying members with a renewed sense of their obligations and their shortcomings.

Animated with a somewhat similar zeal for our mutual welfare, I shall venture this evening to address you briefly upon a matter which deeply concerns us all as professional brethren, and yet which in the hurry of our practical life is easily lost sight of, however important we inwardly admit it to be. Do not be alarmed at the prosiness of the text if I confess to you, without further preface, that the subject of "Character in Professional Life" has seemed to me of sufficiently vital interest to us all as men and as dentists to give me the courage to ask your attention to a passing consideration of its demands upon our lives. I shall most assuredly seek to leave wholly out of the question the morally painful axiom, that character in every department of life and action is a good investment,—that, in fact, it pays. We all know this to be true beyond peradventure; no one is enough of a cynic to attempt to gainsay it; and recognizing this fact, we shall do well never to forget it, and yet never to be sordid enough to urge worldly success as a prime motive for the upbuilding of a strong, persistent, and ever-developing human character. I would appeal to you on far higher grounds to consider with me the essential needs, in our chosen path, of a lofty ideal of manhood, so exalted that it shall put us above even the possibility of pursuing the true course with any other purpose than the supreme satisfaction of having *kept the faith for its own sake*, regardless of personal gain or loss by the way. Sertorius said, "The man who has any dignity of character should conquer with honor, and not use any base means, even to save his life."

I do not propose to give voice to pessimistic comments over the examples—sadly numerous of late years—of men who in many pursuits have set forth apparently with noble intent, who have advanced, seemingly with rectitude and correct standards, and then suddenly have been tempted, have wavered, and then have gone down from high places to the merciful darkness of oblivion,—pitiable failures. It is a commonplace story. You may read it in every daily paper. It hardly excites comment, and an occasional shrug, as we mutter in bitterness and pity, "Another good man gone."

The dangers of a professional life are, fortunately, not so great as those which beset men concerned with vast commercial ventures of the day, where enormous stakes are played for yet more enormous and unjust gains; but the danger differs not in kind, but in degree. Mankind, weak, irresolute, roused only at rare intervals to a quickened sense of the responsibilities of the moral life, has nevertheless a sure, instinctive appreciation for what is better than itself. This has lately been seen in the death recently of a "good, great man" in this city, one who did noble work in behalf of Harvard Dental School, whose one unceasing message to his fellow-men was to impel them to live up to the highest ideals. It will never be adequate to the formation of real character to coax ourselves that we are doing fairly well because debts are paid and a conventional rectitude maintained. Not to personal charm or wealth of intellect will men pay the homage alone due to a majestic character. Any attitude seems preferable, morally, to that sublime conceit which recognizes no necessity nor possibility of advance. This unhappy condition is well reflected in that prayer uttered some years ago by a preacher well known hereabouts, who petitioned his Creator somewhat as follows: "O Lord, now grant us that self-complacency which is the balm of life."

There is little need for me to speak with special significance upon the necessity for medical men of all specialties to keep themselves above the least taint of professional suspicion. Dr. Oliver Wendell Holmes has again and again dwelt, as he so well knows how to dwell, upon the large, generous, yet exacting ethics of the code, which is as binding upon our department of the profession as upon any.

The more obvious side to this question of character, as it concerns us, is the careful avoidance by the slightest word or act of anything which shall call down reproach upon our calling or upon us as men. I do not mean to inculcate, of course, any weak subservience to the dictates of what is called, by convenience, "society,"—that intangible yet forcible consensus of organized humanity which undertakes to say how we shall speak, eat, dress, and behave when in open relations with our fellow-beings. Any timorous sacrifice of personal freedom of action, so long as we know ourselves to be right, is no gain in character. Etiquette—a cultivated and well-sustained courtesy of manners—is, however, by no means despicable. More often than not it is the evidence and the earnest of higher purposes within. Manners are not of necessity morals, but they are the choice fruits of a sound growth. It is certain that those who are of hypocritical purposes will soonest adopt that which they hope will pass for sincerity, by simulating a smooth and winning external conduct, which is in them more repulsive because the genuine thing is so desirable. "Be and continue poor," said Heinzelmann, "while others around you grow rich by fraud and disloyalty; be without place or power, while others beg their way upward; bear the pain of disappointed hopes, while others gain the accomplishment of theirs by flattery; forego the gracious pressure of the hand for which others cringe and crawl. Wrap yourselves in your own virtue, and seek a friend and your daily bread. If you have in your cause grown gray *without unbleached honor*, bless God and die."

With the gradual advancement of softening influences of civilized life many things once admired have had to make way for others still better. One cherished delusion in particular, I fear, will now have to be given up as superannuated. I refer to the man once esteemed among us, with a "kind heart and rough exterior." We are none of us so young that we do not recall the old-fashioned dentist of infancy, of bluff, cheery ways, whose person and office smelled largely of his stable as well as of his pungent drugs. With all the heartiness and moral soundness of these good men, there is now recognized to have been not a little selfishness in their inconsiderate absence of cleanliness and outward deference to others. Their bluffness often was, to the careful observer of human character, a thin cloak for their abnormal sense of their superior knowledge. They lacked, good fellows as they really were, the consequent modesty of the highest wisdom.

Another thing which has already entered largely into our professional life, and which is certain to become a still more prominent influence, is the rapid change of the relations between men and women of the present day. I have not felt it necessary before, gentlemen (as we all surely are here), to dwell upon the imperative call for every one to be solicitous of reputation and to give no occasion for idle speech; nay, rather of that busy speech so ready to undermine the established foundations of a good name, in the foul hope of pulling down the whole structure over its owner's head. It may be difficult to live up to the demands of the highest ethics, but we can at least obey the decalogue. It is not of the grosser forms of misconduct of which there is need to speak, but to call attention simply to the new demands of the age. The now generally observed practice of employment of women assistants in the operating-room of the profession of dentistry we must hold not so much to protect our patients as ourselves. Whenever women co-operate with men in daily life, manners are gentler, courtesy becomes an exchange of mutual consideration, and not a patronizing deference of the stronger to the weaker. The doctor who secures immunity from the too frequent incursions of the sentimental, the hyperæsthetic, or the hysterical patient of the other sex is recognized as a self-respecting man and one who saves time for his practice which he might otherwise, through misunderstandings or malevolence, be forced to devote to explanations, or even as a defendant in a lawsuit. Women—the best sort of women—now wish to be recognized not as women, but as human beings, and there never was a time when frankness, ingenuousness, and honesty of purpose were so well understood. The age of silliness is happily passing in this respect. Dignity, inflexible courtesy to all conditions of age, sex, and of worldly circumstances, are now met with openness and an intelligent appreciation of the motives which underlie outward conduct. Women are quick to see and to approve the spirit of fairness, especially if it be free from all irritating condescension; but before the ideal condition between patient and dentist can be realized, the latter must have definitely shown himself "from spur to plume a stainless knight," no matter what may be the character, the attitude, or the behavior of those whom he may meet, in daily practice. He, at least, must be without fear and without reproach.

Do not for a moment suppose that I am advocating a merely professional manner. Nothing can be more distasteful to those of a delicate perception in morals than that priggish decorum in which the man is wholly merged in the doctor. One must be of poor stuff who does not dare to be himself in any circumstance of life. "Who is that man over there?" asked one man of another. "His name is Higgenbottom, and he looks it." It will not do to *look* professional merely. A man is of some consequence in the world when it is known he can be relied upon; that when he says he will do a thing, he *can* do, and *does* it.

I see one distinct advantage in the possession of a lofty character for those of our avocation, of which it is not easy to speak without exciting, perhaps, derision, and yet, certainly in giving utterance to my thought, I have no wish to provoke even a smile. Doctors, and especially dentists, are frequently made the subjects of many pointless jests as relating to matters financial. Like the lawyer, the doctor and the dentist are often made to feel that the public are fond of regarding themselves as the victims of inconsiderate charges. This is not a pleasant condition to find existing, and yet it undoubtedly does exist, whether as a phase of "American humor" or as a firmly-established theory in the popular mind.

It seems to me that nothing sooner tends to disarm prejudice of this sort than the awakening conviction of those who resort to us, that a professional man who is honorable in most things is apt to be so in all, and that if he respects himself and his character before men, he will of necessity respect the nature, and hence the value, of his professional services. Perhaps this is a trivial matter in your opinion, but at least it is not so to those who approach us for aid. Extortionists and tricksters are responsible for this false estimation in which we are sometimes held, and therefore the more solicitous we are of honor as professional men the more rapidly will the cure operate.

It is said that no member of the callings of medicine and dentistry has ever been elevated to the peerage of England. Many have been knighted, many more have been held in love and veneration, but no one of either profession has ever been offered that prize secretly coveted by every loyal Englishman. The cause is said to lie (though I do not personally know it to be the case) in the deep-rooted prejudice which holds that any one constantly employed in daily contact with the frailties and diseases of humanity is in some sort influenced for the worse by a degrading contamination; in other words, the alleviation of bodily ills, while it may render a man worthy of respect and of a limited recognition, makes it impossible at the same time that he shall be ennobled. It is curious, yet natural withal, with the rapid democratization of life in England, that this feeling is diminishing. Its professional men were never so esteemed, even without its cherished coronets, and, as a logical sequence, they never so much honored themselves or so dignified the callings of dentistry and medicine.

In this country no such unhappy and absurd stigma has ever attached to our position, nor have our opportunities been stifled by social obstructions. A rare courtesy, a dignified and self-centred control, a delicacy and chastity of soul which is probably a part of the national character, has distinguished our profession here and has given it repute abroad.

While we have much cause for self-congratulation, there will always be abundant cause for self-examination and improvement. Born of a race of idealizing tendencies, never to be satisfied with anything less than an absolutely high standard, it becomes us not merely to maintain what is won, but to persist in the arduous path we have so successfully trod. In addition to worldly reputation and to the certainty of personal gain, we must carefully insist upon, for ourselves and for our brethren, a loftiness of character which in political and social life we Americans require in others. Scott's last injunction to his son-in-law was, "Lockhart, I may have but a minute to speak to you. My dear, be virtuous, be religious, be a good man. Nothing else will give you any comfort when you come to lie here."

You will pardon whatever of the commonplace I have uttered this evening. It is needful at times to be very plain. At least, what I have said has been born of conviction.

